# Hypoxia inducible factors regulate infectious SARS-CoV-2, epithelial damage and respiratory symptoms in a hamster COVID-19 model

**DOI:** 10.1371/journal.ppat.1010807

**Published:** 2022-09-06

**Authors:** Peter A. C. Wing, Maria Prange-Barczynska, Amy Cross, Stefania Crotta, Claudia Orbegozo Rubio, Xiaotong Cheng, James M. Harris, Xiaodong Zhuang, Rachel L. Johnson, Kathryn A. Ryan, Yper Hall, Miles W. Carroll, Fadi Issa, Peter Balfe, Andreas Wack, Tammie Bishop, Francisco J. Salguero, Jane A. McKeating

**Affiliations:** 1 Chinese Academy of Medical Sciences Oxford Institute, University of Oxford, Oxford United Kingdom; 2 Nuffield Department of Medicine, University of Oxford, Oxford, United Kingdom; 3 Ludwig institute for Cancer Research, University of Oxford, Oxford, United Kingdom; 4 Radcliffe Department of Surgery, University of Oxford, United Kingdom; 5 Immunoregulation Laboratory, The Francis Crick Institute, London, United Kingdom; 6 United Kingdom Health Security Agency (UKHSA), Porton Down, Salisbury, United Kingdom; University of North Carolina at Chapel Hill, UNITED STATES

## Abstract

Understanding the host pathways that define susceptibility to Severe-acute-respiratory-syndrome-coronavirus-2 (SARS-CoV-2) infection and disease are essential for the design of new therapies. Oxygen levels in the microenvironment define the transcriptional landscape, however the influence of hypoxia on virus replication and disease in animal models is not well understood. In this study, we identify a role for the hypoxic inducible factor (HIF) signalling axis to inhibit SARS-CoV-2 infection, epithelial damage and respiratory symptoms in the Syrian hamster model. Pharmacological activation of HIF with the prolyl-hydroxylase inhibitor FG-4592 significantly reduced infectious virus in the upper and lower respiratory tract. Nasal and lung epithelia showed a reduction in SARS-CoV-2 RNA and nucleocapsid expression in treated animals. Transcriptomic and pathological analysis showed reduced epithelial damage and increased expression of ciliated cells. Our study provides new insights on the intrinsic antiviral properties of the HIF signalling pathway in SARS-CoV-2 replication that may be applicable to other respiratory pathogens and identifies new therapeutic opportunities.

## Author summary

The COVID-19 pandemic, caused by the novel coronavirus SARS-CoV-2, is a global health challenge. While multiple public health approaches including mass vaccination are needed to bring the pandemic under control, there is an urgent need to understand the biology of this virus. This is of particular importance as we learn to live with SARS-CoV-2 as a new endemic disease in the post-vaccine era. Although COVID-19 is mild in most cases, a defining feature of severe disease is systemic low oxygen levels or hypoxaemia. Mammalian cells adapt to low oxygen through an orchestrated transcriptional response regulated by hypoxia-inducible factor (HIF). We previously identified a role for HIFs to inhibit SARS-CoV-2 infection in simple culture systems in the laboratory. We now show that a licensed drug targeting the HIF prolyl hydroxylases inhibits SARS-CoV-2 replication in the Golden Syrian hamster model of infection and ongoing research is investigating the mechanisms responsible for restricting viral replication.

## Introduction

Coronavirus disease (COVID-19), caused by SARS-CoV-2, is a global health issue with more than 5.5 million fatalities to date. Vaccination has reduced both the number of hospitalisations and mortality due to COVID-19 [1, 2]. However, the emergence of variants, such as Omicron, that show reduced sensitivity to vaccine-induced immunity [3–5], provide the potential for new waves of infection. The primary site of SARS-CoV-2 infection is the upper respiratory epithelia with diminishing levels of infection in distal areas of the lung [6]. A defining feature of severe COVID-19 pneumonitis is systemic low oxygen (hypoxaemia), which can lead to organ failure and death through acute respiratory distress syndrome (ARDS) [7, 8]. At the cellular level, hypoxia induces substantial changes to the host transcriptional landscape regulating a diverse array of biological pathways that are orchestrated by hypoxic inducible factors (HIFs). When oxygen is abundant, newly synthesised HIFα subunits are hydroxylated by HIF prolyl-hydroxylase domain (PHD) enzymes resulting in their proteasomal degradation. Under hypoxic conditions the PHD enzymes are inactive and stabilised HIFα dimerizes with HIF-1β, translocates to the nucleus, and promotes the transcription of genes involved in erythropoiesis, glycolysis, pulmonary vasomotor control and immune regulation [9–11]. HIF-target genes can vary between cell types allowing a flexible response to diverse physiological signals [12].

Under normal physiological conditions, the lungs provide an oxygen rich environment, however, an increasing body of literature shows a role for hypoxia in the inflamed airway epithelium [13]. Transcriptomic analysis of post-mortem COVID-19 pulmonary tissue shows an association between hypoxic signalling and inflammatory responses [14, 15]. While HIFs may drive inflammation in certain settings, HIF-1α has been shown to suppress the inflammatory response in bronchial epithelial cells reducing IL-6 and IP10 expression [16]. This dual role for HIFs highlights the importance of the cellular environment in which hypoxia occurs.

HIFs modulate the replication of a wide number of viruses [17], enhancing the replication of hepatitis B [18] and Epstein Barr viruses [19, 20] via direct binding to their viral DNA genomes. In contrast, HIFs inhibit influenza A virus replication in lung epithelial models of infection [21]. These differing outcomes may reflect variable oxygen levels at the site of virus replication in the body. Several respiratory pathogens including Influenza [22], Rhinovirus [23] and Respiratory Syncytial virus [24] induce anaerobic glycolysis via activation of the HIF-1α signalling axis, suggesting a role for viruses to manipulate this pathway. A greater understanding of the oxygen microenvironment in the healthy and inflamed lung will inform our understanding of mucosal host-pathogen interactions.

We reported that hypoxic activation of HIF-1α inhibits SARS-CoV-2 entry and replication in primary and immortalised lung epithelial cells [25]. HIF-1α downregulates the expression of two key entry factors ACE2 and TMPRSS2, thereby limiting SARS-CoV-2 internalisation, whilst also restricting the establishment of viral replication complexes. These data show an essential role for hypoxia/HIF-1α in multiple aspects of the SARS-CoV-2 life cycle and it is timely to address the role of HIFs in an immune competent animal model of COVID-19 disease. We selected to use Golden Syrian hamsters as it represents a well-established and validated COVID-19 model that shows a moderate to severe disease phenotype that is comparable to human infection, including lung pathology and damage to the ciliated epithelia [26–30]. While not all features of severe infection are recapitulated, the susceptibility of hamsters to SARS-CoV-2 infection through the endogenous ACE2 receptor is a benefit when evaluating this step in the virus life cycle. HIFs can be activated by drugs that inhibit the PHDs which are currently used for the treatment of renal anaemia [31–36]. We evaluated the efficacy of the PHD inhibitor FG-4592 (Roxadustat) to inhibit SARS-CoV-2 replication and pathogenesis in Golden Syrian hamsters. Treatment of infected hamsters with FG-4592, either prophylactically or after infection, reduced the infectious viral burden and respiratory symptoms at 4 days post-infection. These findings provide new insights on the interplay between HIF signalling and SARS-CoV-2 replication in an *in-vivo* model that may be applicable to other respiratory pathogens and provide new preventative and therapeutic opportunities.

## Results

### Orally administered FG-4592 activates HIFs in the lung and limits SARS-CoV-2 disease severity

**Fig 1:**
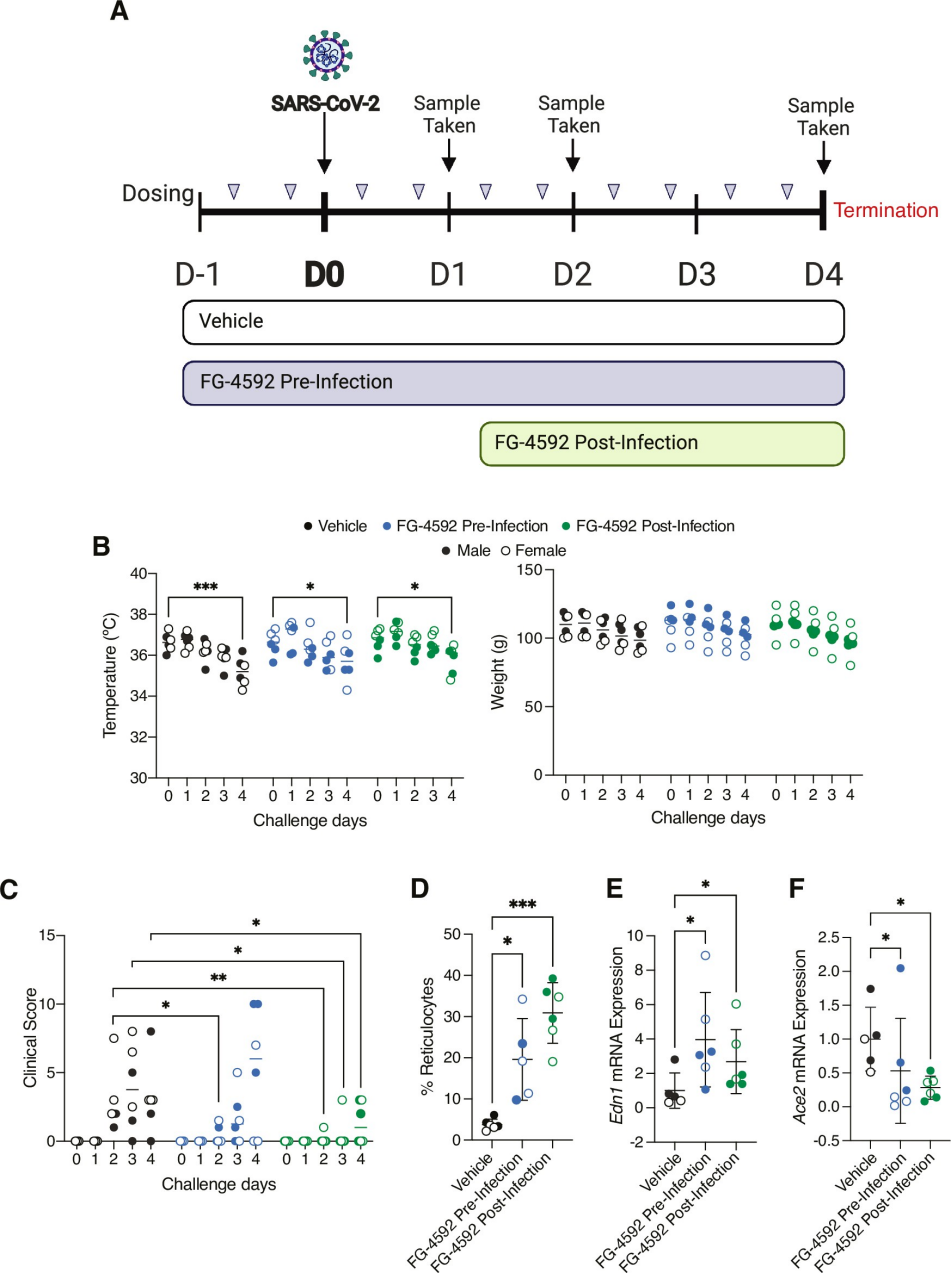
Evaluating FG-4592 in the Syrian hamster model of SARS-CoV-2. **(A)** Schematic of the challenge study. 18 animals aged 7 weeks were allocated into groups of 6 for each treatment. For vehicle and pre-infection groups, twice daily dosing of vehicle or 30mg/kg of FG-4592 administered by oral gavage commenced 24h prior to intranasal challenge with SARS-CoV-2 VIC01/2020 (5x10^4^ PFU). For the post-infection treatment group, FG-4592 dosing started 24h after viral infection. On days 1, 2 and 4 post-infection, nasal washes and throat swabs were collected for assessment of viral load and infectious titre. The study was terminated at 4 days post-infection. **(B)** Hamster temperature and weight measurements over the course of the study. Weights were measured daily, and temperature recorded twice daily with average measurements for each animal plotted. **(C)** Clinical scores from daily animal assessments across the treatment groups. Observations such as wasp waisted, ruffled fur, hunched or laboured breathing were recorded and assigned a numerical value (see **S1 Table**). **(D)** Reticulocyte counts were quantified by staining terminal blood samples with 0.1% Brilliant Cresyl Blue. Endothelin-1 (*Edn1*) **(E)** and *Ace2* **(F)** mRNA levels in the lung and data expressed relative to the mean of the control Vehicle group. Open circles represent female animals and closed circles males. Unless otherwise stated, data is expressed as mean ± s.d. Statistical analysis was performed using a two-way ANOVA, p<0.05 = *, p<0.01 = **, p<0.001 = ***.

To assess the effect of FG-4592 on SARS-CoV-2 infection, hamsters were treated with 30mg/kg of drug twice daily by oral gavage commencing either 24h pre- or 24h post-viral challenge. This regimen was based on previous dosing protocols in mice [25, 37] and clinical studies [38]. In the control group, animals were treated with vehicle 24h prior to infection which continued throughout the study in the same manner as treated animals **(Fig 1A).** Hamsters were infected with SARS-CoV-2 (Australia/VIC01/2020 or VIC01) by intranasal delivery of 5x10^4^ plaque forming units (PFU), which is sufficient to cause clinical signs and respiratory lesions [27, 39, 40]. Weight and body temperature were recorded and clinical signs such as laboured breathing, ruffled fur and lethargy measured twice daily to provide a clinical score (described in **S1 Table**). Infectious virus in the upper respiratory tract was measured in nasal washes and throat swabs collected at 1-, 2- and 4-days post-infection. The study was terminated at 4 days post-infection based on published studies [26, 27, 29] and our in-house experience quantifying infectious virus in the upper respiratory tract **(Fig 1A).**

We observed a significant reduction in body temperature and loss in body weight in all treatment groups **(Fig 1B)**, in line with the clinical presentation of SARS-CoV-2 in this model [26, 27, 41]. No significant differences in temperature or animal weight were noted between the treatment groups, suggesting that FG-4592 was well-tolerated **(Fig 1B).** The first signs of disease were observed at day 2 post-infection, further increasing by day 4 in the control group primarily due to the onset of laboured breathing **(Fig 1C, S1 Table)**. Animals treated with FG-4592 showed a significant improvement in their clinical score, particularly in the post-infection treatment group **(Fig 1C)**. Further, while all animals in the control vehicle group presented with laboured breathing, this was only observed in 2/6 animals in the pre-infection treatment group and none of the hamsters in the post-infection treatment group.

As HIF expression following systemic PHI treatment is transient and difficult to detect [42], we evaluated FG-4592 efficacy by assessing HIF activation of erythropoietin stimulated erythrocytosis by measuring immature red blood cells (reticulocytes). Blood smears from terminal blood samples showed increased reticulocyte counts compared to vehicle, consistent with effective drug treatment **(Fig 1D)**. To evaluate whether FG-4592 activated HIFs in the lung we assessed pulmonary expression of the HIF target gene Endothelin-1 (*Edn-1*) [43] and noted a modest but significant induction of mRNA (**Fig 1E**). Furthermore, we noted a decrease in the transcript levels of the viral entry receptor *Ace2* in the lungs of treated hamsters **(Fig 1F),** supporting our previous findings [25]. To understand the PHI-driven changes in pulmonary gene expression we sequenced RNA from the lung tissue of vehicle, FG-4592 pre- or post- infection groups and observed an induction of 47 and 63 genes respectively, including HIF target genes such as *Edn-1* and *Bnip3* **(S1A Fig)**. To assess whether all animals responded to FG-4592 we evaluated transcript levels of the common HIF-upregulated genes. Hierarchical cluster analysis separated the vehicle and treated animals and showed comparable activation in the pre- and post-infection treatment groups, demonstrating that animals had responded in a similar manner **(S1B Fig)**. Together these data show that FG-4592 is well tolerated, activates HIF-transcriptional responses in the lung and reduces symptoms of SARS-CoV-2 infection.

### FG-4592 reduces infectious SARS-CoV-2 in upper and lower respiratory tract

**Fig 2:**
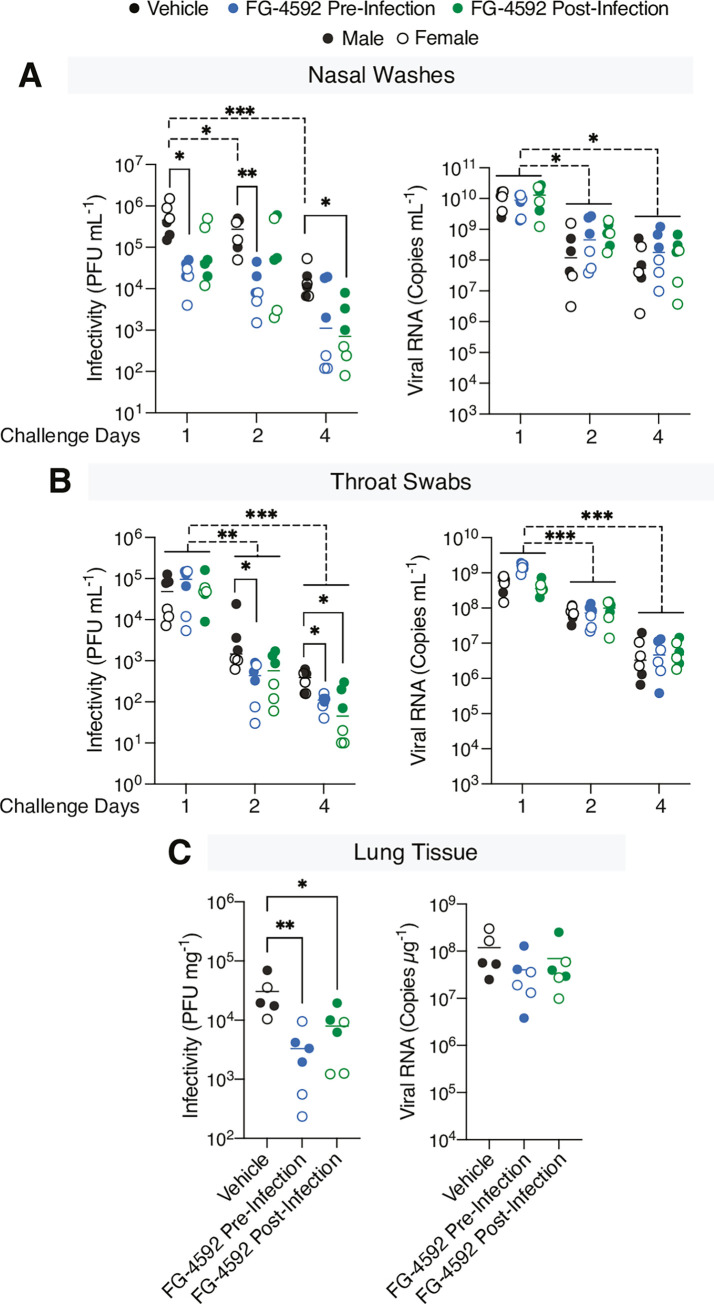
FG-4592 treatment reduces the infectious SARS-CoV-2 burden in the respiratory tract. **(A)** The infectious burden in the nasal washes sampled at days 1, 2, and 4 post-infection was quantified by plaque assay on Vero-TMPRSS2 cells and data presented as plaque forming units (PFU) per ml. Viral RNA copies were measured by RT-qPCR and expressed as copies/mL. **(B)** Viral infectivity and RNA copies were measured in throat swabs as described above. **(C)** Snap frozen lung samples from the right lobe harvested at termination were homogenised for RNA extraction and titration of infectious virus. Viral RNA copies were quantified by RT-qPCR and expressed as copies/μg of total RNA. Infectious titre was determined by plaque assay and expressed as PFU/mg of lung tissue. Open circles represent female animals and closed circles males. Statistical analysis was performed by a two-way ANOVA, p<0.05 = *, p<0.01 = **, p<0.0001 = ***, p<0.0001 = ****. Brackets indicate the comparisons tested.

The course of SARS-CoV-2 disease in the Syrian hamster is transient, with the onset of clinical signs peaking between 4–6 days post-infection followed by the development of neutralising antibodies and viral clearance within 8–15 days [26, 28]. To assess the effect of HIFs on SARS-CoV-2 replication we measured viral RNA by qPCR and infectious virus by plaque assay using Vero-TMPRSS2 cells **(S2 Fig)**. High levels of viral RNA and infectious SARS-CoV-2 were detected in the nasal washes and throat swabs sampled at day 1 post-infection in the vehicle group, which declined over the course of the study **(Fig 2A and 2B)**. Pre-treatment with FG-4592 resulted in a 1-log reduction in the infectious viral burden in both nasal washes and throat swabs at day 2 post-infection **(Fig 2A and 2B)**. Similarly, animals treated post-infection showed significantly reduced levels of infectious virus by day 4. In contrast, drug treatment had a negligible effect on viral RNA levels in either the nasal washes or throat swabs **(Fig 2A and 2B).** Quantifying the burden of infectious virus in the lungs at the end of the study showed a significant reduction in the treated animals (**Fig 2C).** However, there was no substantial change in total or genomic viral RNA (gRNA) **(Figs 2C and S3)**. Together these data demonstrate that PHI treatment before or after infection significantly reduced the infectious viral burden in the upper and lower respiratory tract of infected hamsters.

### FG-4592 reduces SARS-CoV-2 sub-genomic RNAs in the lung

**Fig 3:**
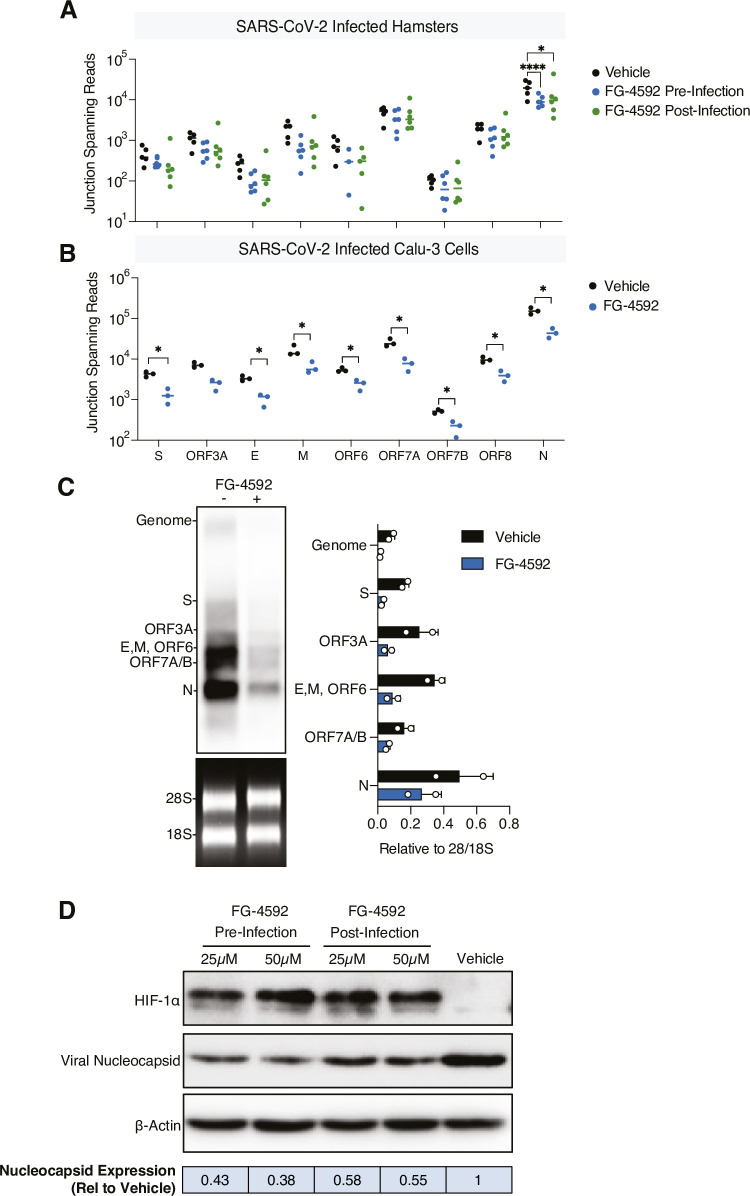
Analysis of the SARS-CoV-2 transcriptome in FG-4592 treated hamster lung and in Calu-3 cells. (A) Viral sequencing reads from infected lung tissue were mapped to the SARS-CoV-2 Victoria reference genome to generate a read depth profile. Reads that spanned the canonical viral sub-genomic transcript junctions were quantified for each tissue sample. **(B)** Junction spanning reads quantified from control or FG-4592 (50μM) pre-treated Calu-3 cells infected with SARS-CoV-2 (MOI of 0.01) (N = 3 biological replicates). **(C)** Viral RNAs from the experiment in (B) were resolved on an RNA-agarose gel and analysed by Northern Blot hybridisation using a dioxygeninin labelled probe designed to detect all viral transcripts. Relative expression of transcripts was determined by densitometric analysis relative to the 28S/18S ribosomal RNA. **(D)** Representative immunoblot of SARS-CoV-2 nucleocapsid (N) and HIF-1α expression in Calu-3 cells treated with FG-4592 (25 or 50μM) pre- or post- infection. N expression was quantified by densitometry relative to β-actin and data expressed relative to the vehicle control. Statistical analysis was performed by ANOVA, p<0.05 = *, p<0.0001 = ****. Brackets indicate the comparisons tested.

Since FG-4592 reduced the level of infectious virus in the lung we were interested to assess whether treatment impacts the viral transcriptome. Mapping the viral reads across the 30kb SARS-CoV-2 genome demonstrated an increasing read depth from ORF1ab to the 3’UTR consistent with the transcription of sub-genomic (sg) RNAs **(S4A Fig).** In addition to the gRNA, the viral transcriptome includes 9 canonical sub-genomic (sg) RNAs that encode the structural proteins, which are essential for the genesis of nascent virus particles. Quantifying the junction spanning reads between the common 5’ leader sequence and start of each sgRNA (as previously described [44]), enabled us to infer their approximate abundance. FG-4592 reduced the abundance of most sgRNAs with a greater variability noted in the treated groups and a significant reduction in the nucleocapsid (N) transcript, the most abundant of the viral RNAs **(Fig 3A)**. We extended these observations to study the effect of HIF-signalling in SARS-CoV-2 transcription in the lung Calu-3 epithelial cell line [45] and showed a significant reduction in S, E, M, ORF6, ORF7A, ORF7B, ORF8 and N junction spanning reads **(Fig 3B)**. The relative abundance of SARS-CoV-2 transcripts were similar in Calu-3 and infected hamster lung tissue. Analysing samples from the infected Calu-3 cells by northern blotting confirmed that FG-4592 treatment reduced viral transcripts **(Fig 3C)**. Furthermore, FG-4592 treatment inhibited N protein expression in infected Calu-3 cells **(Fig 3D)**, providing an explanation for the antiviral activity of HIFs.

Finally, we assessed SARS-CoV-2 sequence variation to determine whether treatment associated with genetic changes. Viral sequences were conserved across the genome in the vehicle or treated lung tissues **(S4B Fig)** and no changes in the consensus sequence were seen in the treated animals or Calu-3 cells, with 100% conservation of the nucleotide sequence across the genome **(S2 Table)**. Together these data show that FG-4592 treatment had no effect on the sequence of SARS-CoV-2 in the lung but reduced sgRNAs.

### Spatial analysis of SARS-CoV-2 RNA and nucleocapsid expression in the respiratory tract

**Fig 4:**
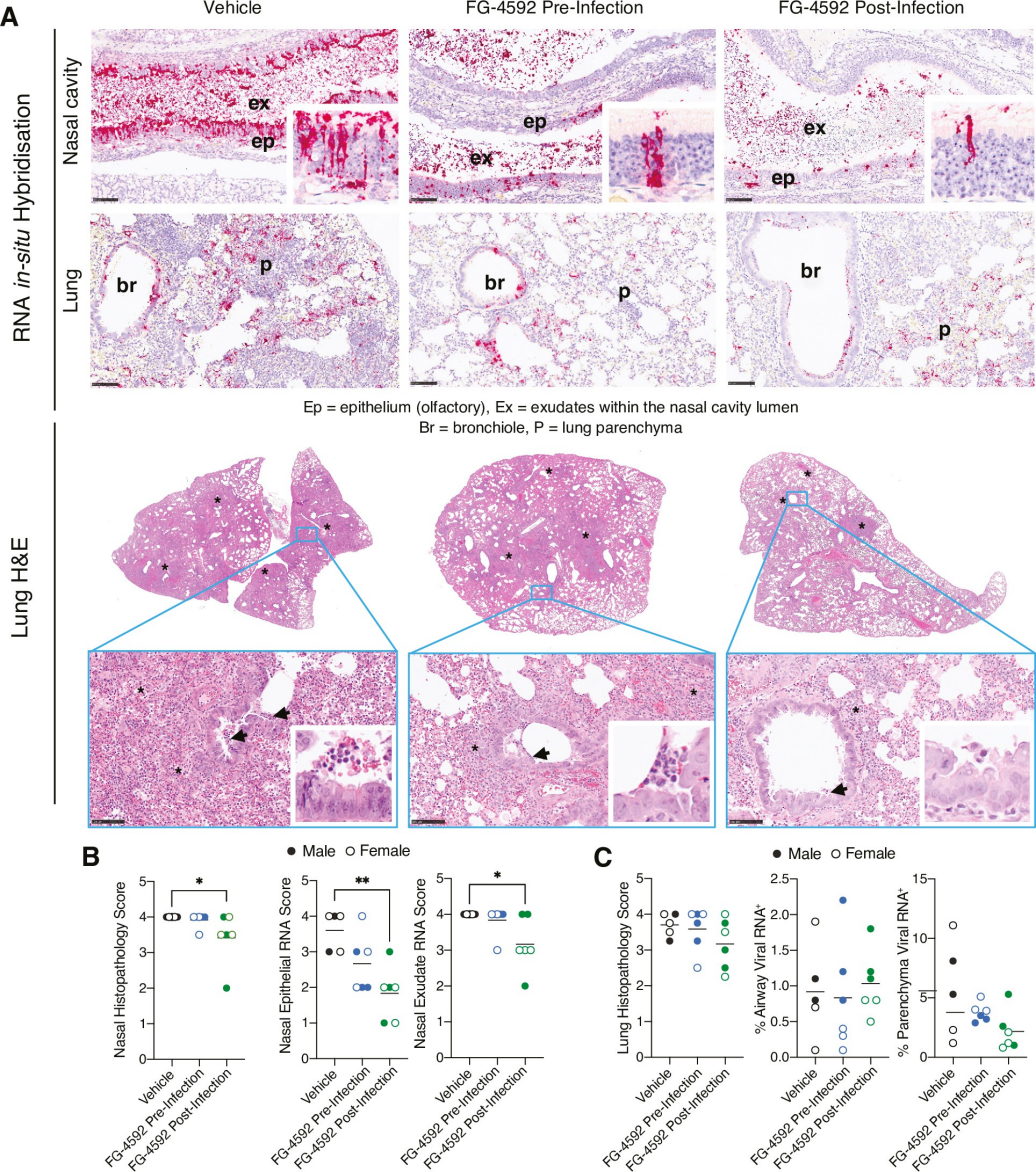
SARS-CoV-2 RNA and pathology in the nasal tract and lung. **(A)** Sections from the left lung and nasal cavity were stained with H&E to analyse histopathological changes and with RNA *in-situ* hybridisation (ISH) to detect SARS-CoV-2 RNA using probes specific for the viral Spike (S) transcript. Representative images from each treatment group are shown. Lung lesions consisted of multifocal broncho-interstitial pneumonia (*) with mild to moderate necrosis of the bronchiolar epithelium (arrows and H&E inserts) (see **S5 Fig**). ISH shows abundant viral RNA in the nasal cavity epithelia that associates with mild to moderate necrosis (inserts) and within the airway epithelia and areas of inflammation. Scale bar = 100μm. **(B)** Nasal cavity was assessed for the presence and severity of lesions using a semi-quantitative scoring system from H&E-stained sections. SARS-CoV-2 RNA in the olfactory epithelium or exudates was quantified using the following scoring system: 0 = no positive staining; 1 = minimal; 2 = mild; 3 = moderate and 4 = abundant staining. **(C)** Lung pathology was assessed for the presence and severity of lesions using a semi-quantitative scoring system from H&E-stained sections. Digital image analysis calculated the area of lung airway and parenchyma staining for viral RNA. Statistical analysis was performed using a one-way ANOVA, p<0.05 = *, p<0.01 = **. N = 5 animals for vehicle and N = 6 animals for each treatment group.

As FG-4592 reduced the clinical signs and levels of infectious virus in the upper and lower respiratory tract we explored the impact of treatment on virus-associated pathology. Sequential sections from the nasal cavity and lung tissue were stained with haematoxylin and eosin (H&E) and with RNA-scope *in situ* hybridization (ISH) probes targeting the Spike gene to assess the tissue distribution of SARS-CoV-2 RNA. We noted extensive inflammatory cell exudate in the nasal cavity and mild to moderate necrosis in both the olfactory and respiratory epithelia **(S5A–S5C Fig)**. We assessed these pathological changes using a semi-quantitative scoring system [46] and showed a reduction in the nasal histopathological score in the post-infection treatment group **(Fig 4B, S3 Table)**. Viral RNA primarily localised to the epithelia and exudate in the nasal cavity and FG-4592 reduced epithelial staining, the major site of virus replication **(Fig 4B)**. A similar observation was noted for viral RNA signals in the exudate **(Fig 4B);** however, these results may be compromised by the daily collection of nasal washes.

**Fig 5:**
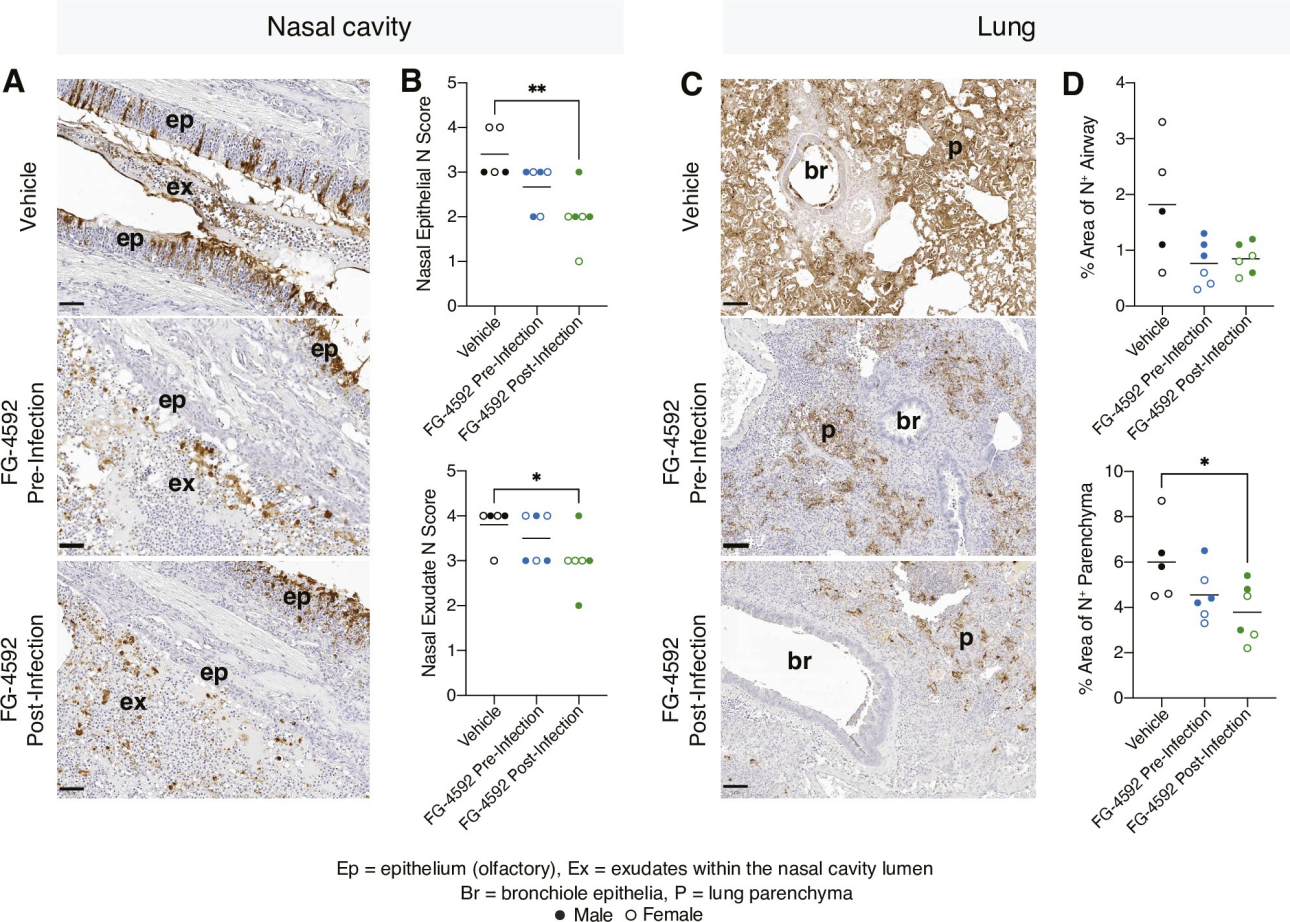
SARS-CoV-2 nucleocapsid expression in the nasal tract and lung. **(A)** Sections from the nasal cavity were stained by immunohistochemistry for the presence of the SARS-CoV-2 N protein. Representative images from each treatment group are displayed. N protein staining was observed in the olfactory epithelia (ep) and the inflammatory exudates (ex) within the nasal cavity luminae. Bar = 50μm. **(B)** A semiquantitative scoring system evaluated N protein expression in the olfactory epithelia or exudates, where: 0 = no positive staining; 1 = minimal; 2 = mild; 3 = moderate and 4 = abundant staining. **(C)** Lung sections were assessed for N protein expression and representative images show positive staining in the bronchiolar epithelia, luminae (br) together and parenchyma (p). **(D)** Digital image analysis calculated the area of lung airway and parenchyma staining for N protein. Statistical analysis was performed by ANOVA, p<0.05 = *, p<0.01 = **. N = 5 animals for vehicle and N = 6 animals for each treatment group.

In agreement with previous studies [41, 46, 47], SARS-CoV-2 infected lung tissue showed pulmonary lesions consisting of broncho-interstitial pneumonia extending into the alveoli and multifocal areas of consolidation, consistent with inflammatory cell infiltration and oedema **(Figs 4A, S5D–S5O)**. Digital image analysis showed that FG-4592 treatment did not alter the severity of lung histopathology **(Fig 4C)**. Viral RNA was detected in the bronchiolar epithelia, bronchiolar inflammatory exudates, as well as in the lung parenchyma of the control vehicle animals **(Fig 4A)** and FG-4592 treatment had no detectable effect on the viral RNA signals in the parenchyma or airways **(Fig 4C)**. To extend these observations we stained the infected nasal and lung sections for SARS-CoV-2 N antigen expression by immunohistochemistry (IHC). Within the nasal cavity, N primarily localised to the epithelia and exudate **(Fig 5A)**, consistent with the detection of S-gene transcripts. Semi-quantitative scoring of the nasal cavity sections showed reduced N antigen expression in the treated animals, most notably in the epithelia of post-infection treated samples **(Fig 5B)**. Within the lung, N staining localised to the airways and lung parenchyma **(Fig 5C)**, and we noted a significant reduction of parenchymal staining in the post-infection treated animals **(Fig 5D)**. In summary, histopathological analysis shows that PHI treatment reduced SARS-CoV-2 RNA and N antigen levels in the nasal epithelia and exudate, consistent with the reduction in infectious viral burden.

### FG-4592 reduces ciliated epithelial damage in the lung

**Fig 6:**
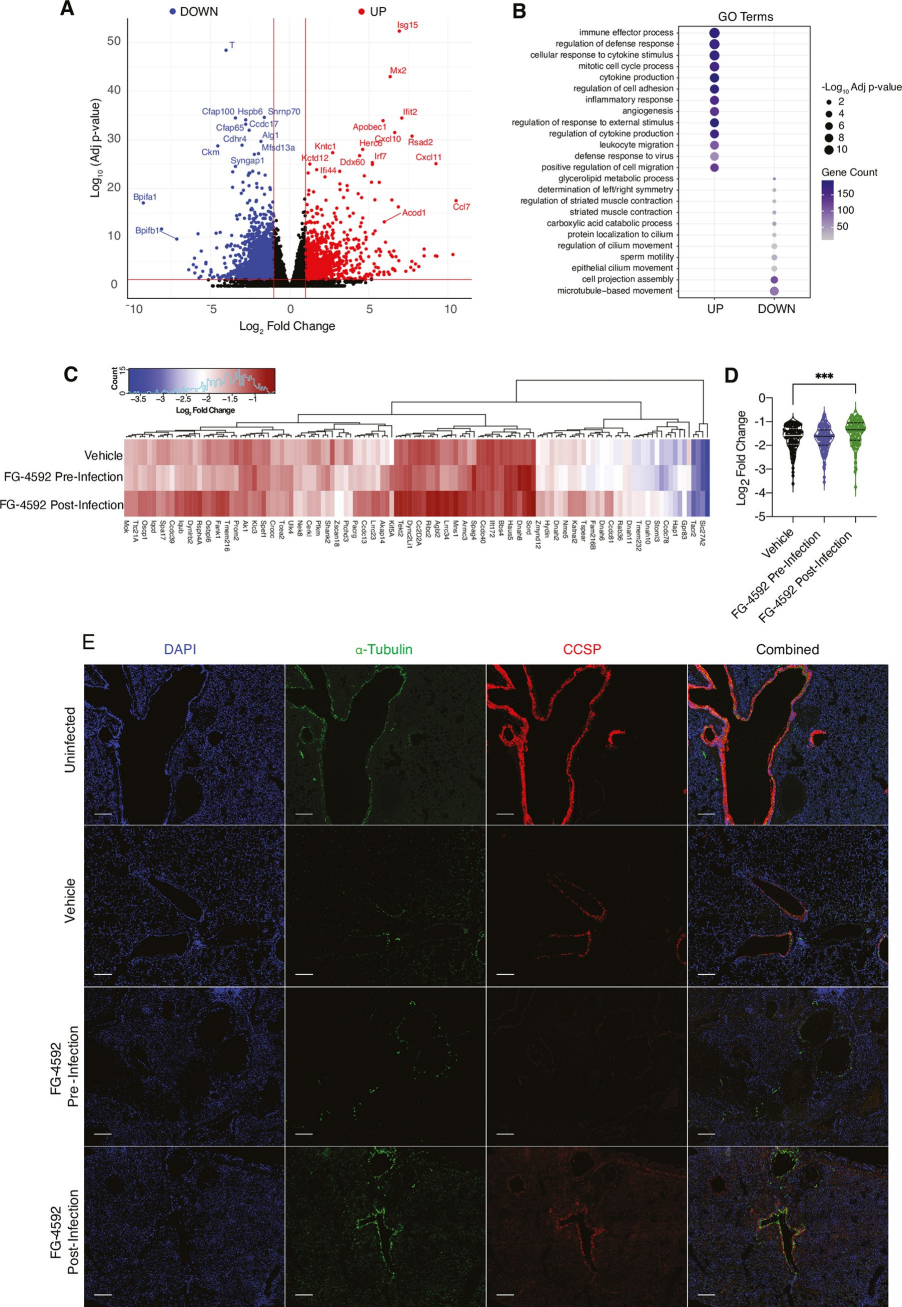
SARS-CoV-2 driven changes in lung ciliated epithelia. (**A**) RNA extracted from snap frozen lung tissue from, vehicle (N = 5), FG-4592 treated pre- (N = 6) and post-infection (N = 6), or uninfected animals (N = 2) was sequenced. Differential gene expression, defined as a log_2_ fold change of +/- 1 with an adjusted p value <0.05, was measured for the SARS-CoV-2 infected vehicle and uninfected samples. (**B**) Significant gene ontology pathways are displayed for both up and down regulated genes in infected vehicle vs uninfected samples, where symbol size reflects the -log10 adjusted p value and gene count is represented by the colour. (**C**) Expression of differentially expressed cilia-related genes, in vehicle, pre- and post-treatment groups compared to uninfected samples. Expression data is grouped by hierarchical clustering. (**D**) Log_2_ fold change values for all genes represented in C and grouped by treatment. Statistical analysis was performed using a one-way ANOVA, *** = p<0.001. (**E**) Representative lung sections stained for α-tubulin or CCSP from uninfected, vehicle, FG-4592 pre-or post-infection treated animals where nuclei are visualised with DAPI. Individual stains are shown along with an overlayed image. Scale bars represent 100μm.

To understand the global host response, we sequenced lung tissue from SARS-CoV-2 infected (vehicle control) and uninfected hamsters. Infection induced substantial changes in the lung transcriptome compared to uninfected tissue; with an up-regulation of pathways involved in inflammation and down-regulation of genes involved in cilium organisation and assembly **(Fig 6A and 6B)**. Analysing inflammatory gene expression using genes from the molecular signature database [48] showed that FG-4592 had a modest effect on the lung inflammasome **(S6 Fig)**. Of note, expression of the viral entry receptors *Ace2* and *Tmprss2* were significantly downregulated (Log_2_ fold change of -1.45 and -1.52 respectively) in the infected tissue, likely reflecting viral cytopathology [14]. Loss of ciliated epithelial cells is a key feature of COVID-19 resulting from damage to the airway epithelia [49–51]. To examine whether FG-4592 reduced the level of epithelial damage we used the reported compendium of cilia-related genes [52] to evaluate gene expression in the pulmonary transcriptome of vehicle and FG-4592 treated animals. We noted comparable patterns of cilia-related gene expression in the vehicle and pre-infection treatment group, with most genes showing a marked down-regulation **(Fig 6C**). However, the viral perturbation of ciliated gene expression was less apparent in animals treated with FG-4592 post-infection **(Fig 6D)**. To gain further insight as to whether FG-4592 treatment affects the level of ciliated cells we stained nasal and lung sections for α-tubulin and club cell secretory protein (CCSP), markers of ciliated cells and secretory cells, respectively. We observed a substantial reduction in α-tubulin and CCSP staining in the infected lung **(Fig 6E)** and nasal cavity **(S7 Fig)**, consistent with virus-induced loss of ciliated cells **(Fig 6E)**. While limited staining was observed in the lung sections from the pre-treated animals, we noted a restoration of α-tubulin expression in post-infection treated sections, in line with our transcriptomic analysis. To establish whether FG-4592 affected cell proliferation we measured Ki-67 expression in lung sections and no significant changes were observed in both treatment groups **(S8 Fig)** Together these data show that a loss of ciliated cells in the respiratory tract is a prominent feature of SARS-CoV-2 infection and FG-4592 treatment may offer some protection from this severe pathological change.

## Discussion

In this study we evaluated the antiviral potential of FG-4592 in the Syrian hamster model of SARS-CoV-2 infection. Treating animals pre- or post-infection reduced the levels of infectious virus and improved clinical signs. The drug was well tolerated, with no adverse reactions reported in any of the treated animals. Despite treatment showing a significant reduction in the levels of infectious virus, bulk PCR quantification of viral RNA in the nasal washes and throat swabs were unchanged. An earlier study of SARS-CoV-2 infection in Syrian hamsters reported a relatively short contagious period that associated with the detection of infectious virus [46]. However, SARS-CoV-2 RNA can persist in the respiratory tract long after the communicable period has passed [26, 28, 46]. SARS-CoV-2 RNA genomes are highly structured, and this may contribute to their persistence [53, 54]. Several publications have reported a discrepancy between viral RNA levels and the detection of infectious virus in clinical samples [55–57]. While quantitative PCR measurement is the gold standard for SARS-CoV-2 diagnosis, this method only detects the viral nucleic acid and not the infectious capacity of the virus particles. It’s relevant to consider the cellular location of the viral RNA in the respiratory tract, where ISH probing of infected nasal tissue revealed SARS-CoV-2 RNA in both the nasal exudate and epithelia. We hypothesise that the exudate comprises extracellular encapsidated viral RNA and processed viral particles in immune cells and does not reflect sites of active virus replication. Importantly, our *in-situ* analysis highlights a significant reduction of viral RNA in the nasal epithelia of FG-4592 treated animals, demonstrating antiviral drug activity at the primary site of replication.

While the disconnect between viral RNA levels and infectivity is not well understood in a clinical setting, we would predict that drugs lowering the levels of infectious virus in the upper respiratory tract will reduce virus transmission. The approved anti-viral drug Molnupiravir showed a negligible effect on viral RNA or infectious titre in respiratory samples when tested in Syrian hamsters [58]. Yet both Molnupiravir and FG-4592 treatments were associated with significant reductions in the burden of infectious virus in the lung. While both drugs have contrasting mechanisms of action, the ability of FG-4592 to limit viral replication highlights the value of targeting host pathways that are essential for viral replication in concert with developing direct-acting antiviral (DAA) agents. A promising area for future development is a combined treatment of PHI and DAAs such as Molnupiravir or the protease inhibitor Nirmatrelvir [59]. Earlier trials of DAA monotherapies for the treatment of HIV or HCV showed the rapid selection of drug resistant viruses, reinforcing the value of combination therapies that provide a higher barrier to the development of anti-viral resistance [60]. Our analysis of the SARS-CoV-2 sequences show no evidence of mutational change in treated animals. However, the viral transcriptome in the lung showed differences in the abundance of sgRNAs in the treated animals, similar to our observations with infected Calu-3 cells where FG-4592 significantly reduced sgRNAs and N protein expression. These data show a role for HIFs in regulating SARS-CoV-2 sgRNA levels that could be explained by changes in the genesis or maintenance of viral replication complexes, in line with our previous observations [25].

An important finding of this study was the improved clinical score in the treated animals. FG-4592 substantially reduced the incidence of laboured breathing in the infected animals, irrespective of treatment grouping, that may be attributable to the increased levels of erythropoiesis resulting in improved blood oxygenation in the infected hamsters. HIFs have been reported to enhance endogenous pathways to protect lung injury during acute inflammation that include: promoting vascular repair [61]; regenerating alveolar type II pneumocytes [62]; preventing the bioenergetic failure of alveolar epithelial cells during ARDS [63]; interacting with key metabolites such as succinate to limit acute lung injury damage [64]. Taken together, these studies implicate HIF activation as a therapeutic strategy to combat lung injury during ARDS and ongoing clinical trials are evaluating the use of PHIs for treating ARDS in the context of COVID-19 (ClinicalTrials.gov Identifier: NCT04478071). Indeed, these mechanisms of endogenous lung protection may explain why most patients with COVID-19 do not develop ARDS.

Histopathological analysis of pulmonary tissue showed that a short duration of FG-4592 treatment improved virus-induced pathology in the upper respiratory tract. Lungs from the treated animals showed fewer areas of consolidation and pulmonary lesions, particularly in the post-infection treatment group. An earlier study reported that SARS-CoV-2 induced lung pathology in this model peaked at 5 days post-infection, after which tissue injury recovered along with viral clearance [27]. Since the primary goal of this study was to evaluate the effect of PHIs on viral replication rather than pathological changes, we predict that longer PHI treatment times would have more pronounced effects on pulmonary pathology and potentially improve recovery times. Furthermore, a substantial body of evidence shows that HIFs can protect from severe lung injury via the induction of extracellular nucleotide metabolism and adenosine signalling [65]. HIF-1α driven expression of the *Adora2b* adenosine receptor in alveolar epithelial cells and subsequent activation of adenosine signalling [66] can protect mice from excessive inflammation during acute lung injury [67–69].

A key feature of SARS-CoV-2 infection is the profound loss of the ciliary layer in the respiratory tract [49–51]; induced either by direct infection of these cells and their subsequent dedifferentiation [49] or loss by cytopathic infection. The resulting impairment in mucociliary clearance limits the removal of infiltrating viral, bacterial, or fungal pathogens that can lead to secondary infections with antibiotic resistant strains of *Staphylococcus aureus* and *Klebsiella pneumoniae* [70], the incidence of which markedly increases in critically ill patients [70, 71]. Our lung transcriptomic analysis highlighted the altered expression of genes involved in ciliated cell function in FG-4592 treated animals. Components of the centrosome, important for cilia formation, are regulated by PHDs and hypoxia increases their expression [72], providing a potential mechanism for our observation. Nonetheless, we are unable to assess whether treatment prevents infection of ciliated cells or promotes their recovery. Staining tissue sections for α-tubulin expression, a marker for ciliated cells, in the upper and lower respiratory tract shows the considerable impact of infection on the abundance of ciliated cells. Importantly, lung sections from animals treated with FG-4592 post-infection showed elevated levels of ciliated cell staining, that may reflect differences in the transcriptional response of infected cells to PHIs. Since there were no detectable changes in expression of the proliferation marker Ki-67 in the lungs of the treated animals, FG-4952 is unlikely to alter the proliferative status of the ciliated epithelia. Healthy cells will trigger reactions enabling them to adapt to hypoxia such as switching from oxidative phosphorylation to glycolysis [73], however, SARS-CoV-2 infection may alter the cellular response to hypoxia potentially exacerbating necrosis, cytokine expression and inflammatory responses [74].

There are limited therapeutic options for treating COVID-19 most of which treat the clinical manifestation of the disease. FG-4592 treatment before or after SARS-CoV-2 infection reduced the infectious viral burden and restored the loss of ciliated cells, providing an opportunity to improve clinical outcomes by limiting secondary infections. Our observations may be applicable for the treatment of other respiratory pathogens, including both Influenza A virus [21, 22] and Respiratory Syncytial virus, whose replication has been reported to be HIF-dependent [75]. In summary, we demonstrate a role for HIFs to suppress SARS-CoV-2 infection and associated disease in an experimental animal model, highlighting the value of prolyl-hydroxylase inhibitors for the treatment of COVID-19.

## Methods

### Ethics statement

All experimental work was conducted under the authority of a UK Home Office approved project licence that had been subject to local ethical review at Public Health England (now part of the UK Health Security Agency (UKHSA) Porton Down by the Animal Welfare and Ethical Review Body (AWERB) as required by the Home Office Animals (Scientific Procedures) Act 1986. The study was performed under the authority of a UK Home Office approved project licence (reference PDC57C033).

### Animals

Golden Syrian hamsters (*Mesocricetus auratus*) aged 6–8 weeks were obtained from Envigo RMS UK Ltd., Bicester, UK. The animals were housed in cages that are designed in accordance with the requirements of the UK Home Office Code of Practice for the Housing and Care of Animal Used for Scientific Procedures (1986). During procedures with SARS-CoV-2, the animals were housed in a flexible-film isolator within a Containment Level 3 facility. The animals were randomly assigned into groups and individually housed, with equal allocation of male and female animals to each study. For direct intranasal challenge studies, group sizes of 6 hamsters were used as the minimal number required for statistical significance to be achieved. Access to food and water was ad libitum and environment enrichment was provided. Rooms were maintained within set parameters: 20–24°C, 45–65% humidity and a 12/12 light cycle.

### Syrian Hamster study design and ethics approval

Animals were divided into three groups for treatment with vehicle, FG-4592 pre-infection or post-infection (n = 6 per group). Animals were treated with 30mg/kg of FG-4592 (MedChem Express) by oral gavage. Drug was dissolved in 99% double distilled H_2_O, 0.5% methyl cellulase and 0.5% Tween-80, administered twice daily. Treatment commenced either 24h prior to (pre) or 24h following (post) infection and maintained until termination of the study (4 days) after infection. The control group followed the dosing schedule of the pre-infection group and were treated with vehicle only. All groups were infected intranasally with 5x10^4^ PFU of Australia/VIC01/2020 SARS-CoV-2. Viral inocula were made in sterile phosphate buffered saline (PBS) and delivered via intranasal instillation (200μL total with 100μL per nare) with animals sedated using isoflurane. Animal weights and temperatures were monitored daily and visual inspection of all animals carried out twice daily, with signs of clinical disease such as wasp waisted, ruffled fur, hunched or laboured breathing recorded (**S1 Table**). Throat swabs and nasal washes were collected on days 1, 2 and 4 post infection. Animals were euthanised at day 4 post infection and tissues collected at necropsy for pathology and virology assays.

### Virus and cells

SARS-CoV-2 Australia/VIC01/2020[76] was provided by the Peter Doherty Institute for Infection and Immunity, Melbourne, Australia at P1 and passaged twice in Vero/hSLAM cells (Cat#04091501) obtained from the European Collection of Cell Cultures (ECACC), UK. Virus infectivity was determined by plaque assay on Vero-TMPRSS2 cells as previously reported [25]. Calu-3 cells were obtained from Prof Nicole Zitzmann’s lab and maintained in Advanced DMEM, 10% FCS, L-glutamine and penicillin streptomycin. Calu-3 cells were infected with the above strain of SARS-CoV-2 at an MOI of 0.01 for 2h. Viral inocula were removed, cells washed three times in PBS and maintained in growth media until harvest.

### Plaque assay quantification of virus infectivity

Samples from nasal washes, throat swabs or lung homogenates were serially diluted 1:10 and used to inoculate monolayers of Vero-TMPRSS2 cells for 2h. Inocula were removed and replaced with DMEM containing 1% FCS and a semi-solid overlay consisting of 1.5% carboxymethyl cellulose (SIGMA). Cells were incubated for 72h, after which cells were fixed in 4% PFA, stained with 0.2% crystal violet (w/v) and visible plaques enumerated.

### qPCR quantification

Viral RNA was extracted from nasal washes or throat swabs using the QiaAMP Viral RNA kit (Qiagen) according to manufacturer’s instructions. Tissues were homogenised using the GentleMACS homogeniser in RLT buffer and extracted using the RNeasy kit (Qiagen) according to manufacturer’s instructions. For quantification of viral or cellular RNA, equal amounts of RNA, as determined by nanodrop, were used in a one-step RT-qPCR using the Takyon-One Step RT probe mastermix (Eurogentec) and run on a Roche Light Cycler 96. For quantification of viral copy numbers, qPCR runs contained serial dilutions of viral RNA standards. Total SARS-CoV-2 RNA was quantified using: 2019-nCoV_N1-F: 5’-GAC CCC AAA ATC AGC GAA AT-3’, 2019-nCoV_N1-R: 5’-TCT GGT TAC TGC CAG TTG AA TCT G-3’, 2019-nCoV_N1-Probe: 5’-FAM-ACC CCG CAT TAC GTT TGG TGG ACC-BHQ1-3’. Genomic viral RNA was quantified using SARS-CoV-2-gRNA_F: 5’- ACC AAC CAA CTT TCG ATC TCT TGT-3’, SARS-CoV-2-gRNA_R: 5’-CCT CCA CGG AGT CTC CAA AG-3’, SARS-CoV-2-gRNA_Probe: 5’ FAM-GCT GGT AGT GAC TGC TTT TCG CCC C-BHQ1-3’. Hamster host transcripts were quantified using the following Taqman expression assays by ThermoFisher, *Edn1* (APAAFZZ), *Ace2* (1956514) and *β-Actin* (APZTJRT).

### Histopathology, *in situ* hybridisation and immunohistochemistry

The nasal cavity and left lung were fixed by immersion in 10% neutral-buffered formalin and processed into paraffin wax. Nasal cavity samples were decalcified using an EDTA-based solution prior to longitudinal sectioning to expose the respiratory and olfactory epithelium. Sequential 4 μm sections were stained with H&E. In addition, samples were stained using the *in-situ* hybridisation RNAscope technique to label SARS-CoV-2 RNA using V-nCoV2019-S probe (Cat No. 848561, Advanced Cell Diagnostics). Briefly, tissues were pre-treated with hydrogen peroxide for 10 min (room temperature), target retrieval for 15 min (98–101°C) and protease plus for 30 min (40°C) (Advanced Cell Diagnostics). The probe was incubated with the tissues for 2h at 40°C and the signal amplified using RNAscope 2.5 HD Detection kit–Red (Advanced Cell Diagnostics). Immunohistochemical (IHC) staining of the SARS-CoV-2 nucleocapsid (N) protein, deparaffinisation and heat-induced epitope retrieval were performed on the Leica BOND-RXm using BOND Epitope Retrieval Solution 2 (ER2, pH 9.0) for 30 minutes at 95°C. Staining was performed with the BOND Polymer Refine Detection kit, a rabbit anti-SARS-CoV-2 nucleocapsid antibody (Sinobiological; clone: #001; dilution: 1:5000) and counterstained with haematoxylin. IHC staining of Ki67 was performed with the same protocol using a rabbit polyclonal anti-Ki67 primary antibody (Abcam; ab15580; dilution 1:500; pH6 antigen retrieval for 20mins). The H&E, ISH and IHC stained slides were scanned using a Hamamatsu S360 digital slide scanner and examined using ndp.view2 software (v2.8.24). Lung tissue from one animal in the vehicle group was not processed due to deterioration of the sample. Digital image analysis using Nikon NIS-Ar software quantified SARS-CoV-2 RNA, N, or Ki67 expression in the lung sections by calculating the percentage of positively stained areas in defined regions of interest (ROI), including the airway epithelia and parenchyma. For the nasal cavity a semi-quantitative scoring system [46] evaluated the presence of SARS-CoV-2 RNA, N or Ki67 expression in the exudate and epithelia where: 0 = no staining; 1 = minimal; 2 = mild; 3 = moderate and 4 = abundant staining. All slides were evaluated subjectively by a qualified pathologist, blinded to treatment details, and were randomised prior to examination to limit bias (blind evaluation). Random slides were peer-reviewed by a second pathologist. Histopathology was carried out in a ISO9001:2015 and GLP compliant laboratory. Digital image analysis quantified the percentage of the lung section area that showed pneumonia. Moreover, a semiquantitative scoring system evaluated the severity of lesions in the lung and nasal cavity as previously reported [46]. Lung sections were scored using the following parameters: a) presence of airway epithelial degeneration; b) necrosis and/or inflammatory cell infiltrates; c) peri-airway inflammatory infiltrates; d) perivascular cuffing, and e) infiltration of alveolar walls by inflammatory cells. In the nasal cavity, two parameters were used in the scoring system: a) epithelial attenuation/necrosis in the mucosa and b) presence of exudates in the nasal cavity lumen. For each parameter, scores were given as 0 = within normal limits; 1 = minimal; 2 = mild; 3 = moderate and 4 = marked/severe.

### RNA sequencing and data analysis

RNA was extracted from 30mg of homogenised lung (right lobe) using the RNeasy kit (Qiagen) and RNA integrity determined by Tapestation (Agilent), before providing RNA to Novogene UK Ltd for poly-A-enriched transcriptome sequencing. Paired end Illumina sequencing was carried out with a 300bp fragment length and mapped to the *Mesocricetus auratus* genome. Viral reads were mapped to the SARS-CoV-2 reference genome (NC 045512.2) using Salmon [77]. FPKM values were enumerated, and differential expression quantified using the DeSeq2 package [78]. Threshold for statistical significance was set as log_2_FC +/- 1 and an adjusted p value <0.05. Junction spanning reads were detected as described in Kim et al 2021[44] using the ggsashimi analysis package [78].

### Immunoblotting

Cells were prepared by washing cells with PBS and lysed using RIPA buffer (20 mM Tris, pH 7.5, 2 mM EDTA, 150 mM NaCl, 1% NP40, and 1% sodium deoxycholate) supplemented with protease inhibitor cocktail tablets (Roche). Clarified samples were mixed with laemmli sample buffer, separated by SDS-PAGE and proteins transferred to polyvinylidene difluoride membrane. Membranes were blocked in 5% milk in PBS/0.1% Tween-20 and incubated with anti-HIF-1α (BD Biosciences), anti-β-Actin (Sigma) or SARS-CoV-2 nucleocapsid (EY-2A, a kind gift from Prof Alain Townsend) primary antibodies and appropriate HRP-conjugated secondary antibodies (DAKO). Chemiluminescence substrate (West Dura, 34076, Thermo Fisher Scientific) was used to visualize proteins using a G:Box Imaging system (Syngene).

### Visualising SARS-CoV-2 RNAs by Northern Blotting

Infected Calu-3 cells were harvested in Trizol (Thermofisher) 24h post infection and total RNA extracted according to manufacturer’s instructions. 10 μg of RNA was resolved on a 10% MOPS, 2.2 M formaldehyde agarose gel. To show equal RNA loading, the 18S and 28S ribosomal subunit RNA species were visualised under UV light through ethidium bromide staining. Gels were denatured in 50 mM NaOH for 5 minutes, and RNAs transferred to nylon membrane by capillary transfer in 20X SSC buffer. Membranes were washed and RNAs fixed by UV crosslinking. Membranes were hybridised at 65°C overnight with a digoxigenin-labelled DNA probe specific to the 3’ end of the SARS-CoV-2 genome, enabling the detection of all viral RNAs. Bands were visualised using a luminescent DIG detection kit (Roche) according to manufacturer’s instructions.

### Statistical analysis

All data are presented as mean values ± SEM. P values were determined using the Mann-Whitney test (two group comparisons) or with the Kruskal–Wallis ANOVA (multi group comparisons) using PRISM version 8. In the figures * denotes p < 0.05, ** < 0.01, *** <0.001 and **** <0.0001.

## Supporting information

S1 Fig FG-4592 induced gene expression in the lungs of SARS-CoV-2 infected hamsters. (A) RNA extracted from frozen lung tissue from each of the three treatment groups, vehicle (n = 5), FG-4592 pre-infection (n = 6) and FG-4592 post-infection (n = 6) was subjected to whole RNA sequencing. Differential gene expression, defined as a log_2_ fold change of -/+ 1 with an adjusted p value <0.05, was assessed between vehicle vs pre-infection or post-infection treatment groups. (B) Heatmap showing the FPKM values of 21 differentially expressed genes common to both pre- and post-infection FG-4592 treatment for each animal grouped by hierarchical clustering analysis. Histogram in legend represents the distribution of values in total data set.
(PDF)

S2 Fig Vero vs Vero-TMPRSS2 in SARS-CoV-2 Plaque Assay. Vero, or Vero-TMPRSS2 cells were infected with a 10-fold serial dilution of SARS-CoV-2 (VIC-01/20) for 2h prior to addition of the semi-solid overlay. Plates were fixed at 24, 48, and 72h post infection and stained with crystal violet to visualise plaques. PFU ml^-1^ values for Vero and Vero-TMPRSS2 cells were estimated to be 3x10^6^ and 2x10^6^ PFU ml^-1^ respectively. These values were calculated using the earliest timepoint plaques were visible.
(PDF)

S3 Fig Quantification of genomic SARS-CoV-2 RNA in the lung tissue. SARS-CoV-2 genomic RNA was quantified from total RNA extracted from the lung tissue of infected hamsters by qPCR using a primer-probe set specific for the viral ORF1A/B region. Open circles represent female animals and closed circles males.
(PDF)

S4 Fig SARS-CoV-2 sequence analysis from hamster lung tissue. (A) SARS-CoV-2 viral reads were mapped to the genome using the SALMON alignment package and read depth at each position in the genome quantified. Samples from individual animals are grouped by the treatment they received. (B) Variability of the viral sequence across the treatment groups. At each nucleotide position the percentage of reads for each nucleotide (A, C, T, G) was divided by the read depth so that a value of 0 would represent 100% conservation across all reads.
(PDF)

S5 Fig Lung and Nasal Cavity Histopathology. Histopathological changes observed in the nasal cavity (A-C) and lung (D-O). The main changes observed in the nasal cavity are presence of abundant mixed inflammatory exudates in the lumen (*) and mild to moderate epithelial cell necrosis (arrows) (A-C). Epithelial cell necrosis is observed in the bronchiolar epithelium (arrows) (D-F). Alveolar changes include presence of inflammatory cells within the alveolar spaces (*) and oedema (arrows) (G-I), together with type II pneumocyte proliferation (J-L; arrows). Perivascular cuffing with presence of inflammatory cells surrounding blood vessels (arrows) is observed (M-O). Scale bar = 50 μm.
(PDF)

S6 Fig The inflammatory response in FG-4592 treated lung samples. Expression of the inflammatory hallmark genes derived from the MSig database in vehicle, pre- and post-infection treatment with FG-4592 compared to uninfected samples. Hierarchical clustering was used to group genes according to expression.
(PDF)

S7 Fig Ciliated cells in the nasal cavity. Representative nasal cavity sections from uninfected, vehicle, pre-infection, or post infection FG-4592 treated animals were stained by immunohistochemistry for a-tubulin and CCSP with nuclei visualised by DAPI. Individual stains are shown along with an overlayed image. Scale bars represent 100μm.
(PDF)

S8 Fig Ki67 staining in the lung. Lung sections from vehicle, pre- and post-infection FG-4592 treated animals were stained for Ki67 by immunohistochemistry. Br = bronchiole, p = parenchyma. Images were subjected to digital image analysis to quantify the amount of Ki67 staining as a percentage of the total area of either airway or parenchyma. Scale bar = 50 μm. Open circles represent female animals and closed circles males.
(PDF)

S1 Table Clinical Observations. (XLSX)

S2 Table Multiple Sequence Alignment of Viral Reads. (XLSX)

S3 Table Hamster Pathology Scores. (XLSX)
