# Supplementary figures and images for: Hypoxia inducible factors regulate infectious SARS-CoV-2, epithelial damage and respiratory symptoms in a hamster COVID-19 model
Source: PLoS Pathog. 2022 Sep 6;18(9):e1010807. doi: 10.1371/journal.ppat.1010807 (PMC9481176; doi:10.1371/journal.ppat.1010807)

**A**

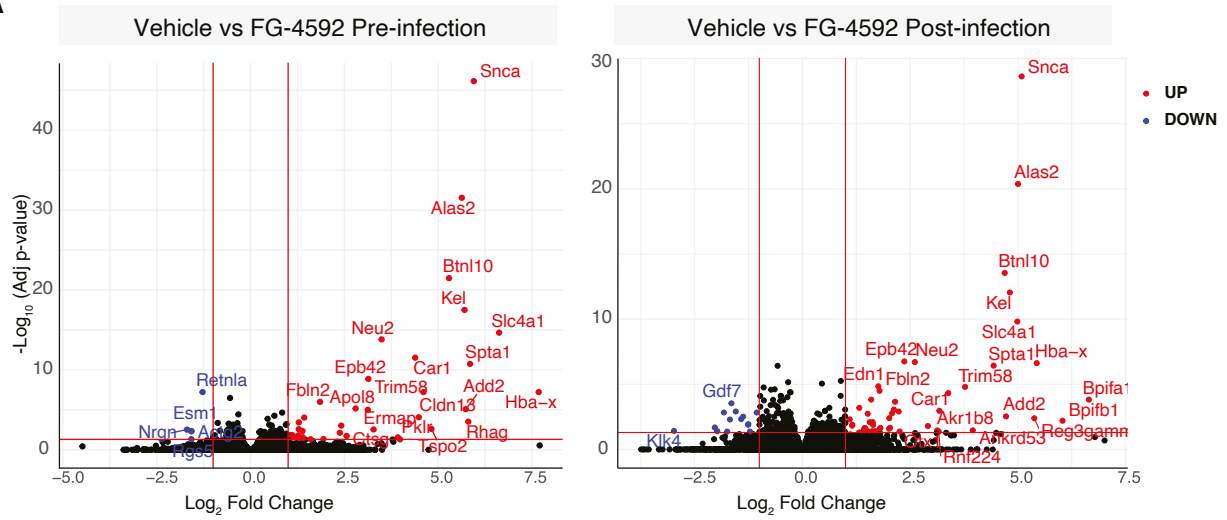

**B**

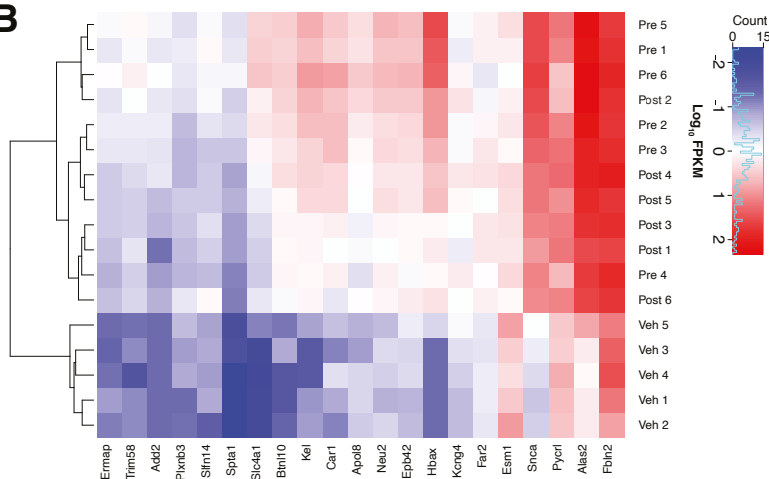

Supplement: S1 Fig — (A) RNA extracted from frozen lung tissue from each of the three treatment groups, vehicle (n = 5), FG-4592 pre-infection (n = 6) and FG-4592 post-infection (n = 6) was subjected to whole RNA sequencing. Differential gene expression, defined as a log2 fold change of -/+ 1 with an adjusted p value <0.05, was assessed between vehicle vs pre-infection or post-infection treatment groups. (B) Heatmap showing the FPKM values of 21 differentially expressed genes common to both pre- and post-infection FG-4592 treatment for each animal grouped by hierarchical clustering analysis. Histogram in legend represents the distribution of values in total data set. (PDF) [file ppat.1010807.s001.pdf]

### Vero

Dilution of  
SARS-CoV-2

24h

48h

72h

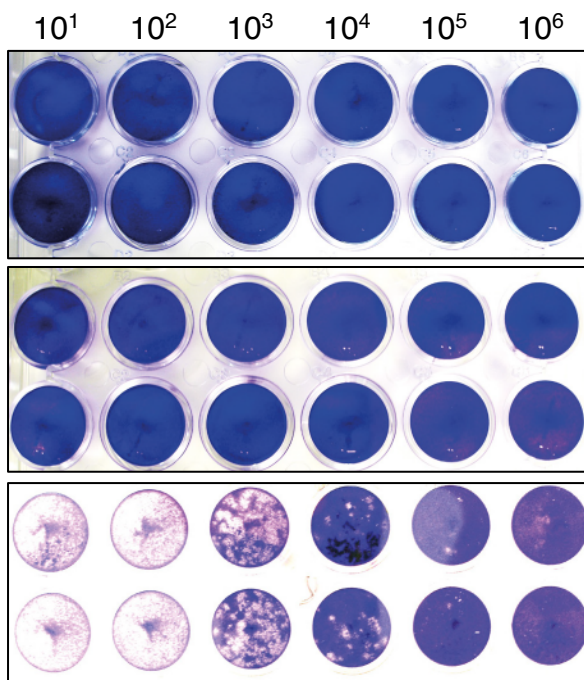

### Vero-TMPRSS2

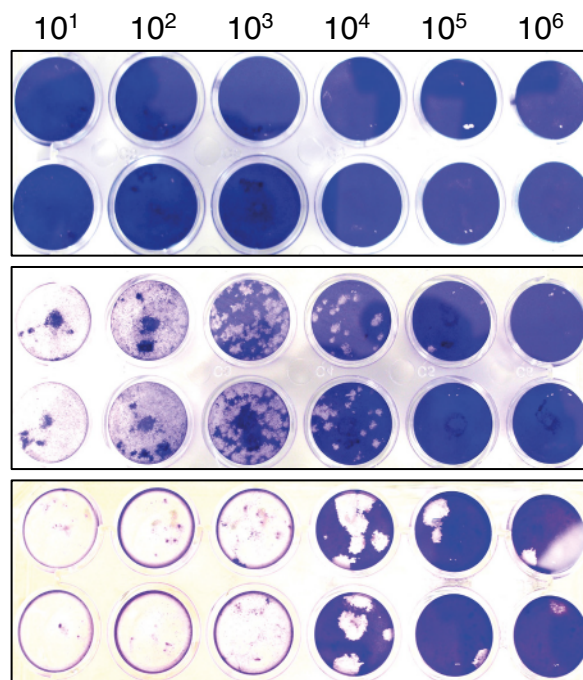

Supplement: S2 Fig — Vero, or Vero-TMPRSS2 cells were infected with a 10-fold serial dilution of SARS-CoV-2 (VIC-01/20) for 2h prior to addition of the semi-solid overlay. Plates were fixed at 24, 48, and 72h post infection and stained with crystal violet to visualise plaques. PFU ml-1 values for Vero and Vero-TMPRSS2 cells were estimated to be 3x10^6^ and 2x10^6^ PFU ml-1 respectively. These values were calculated using the earliest timepoint plaques were visible. (PDF) [file ppat.1010807.s002.pdf]

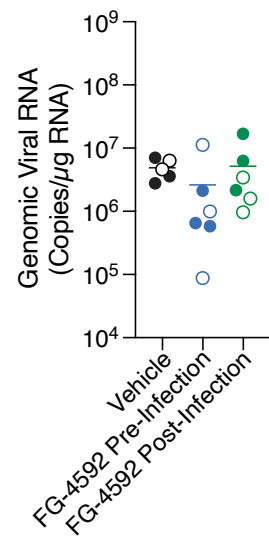

Supplement: S3 Fig — SARS-CoV-2 genomic RNA was quantified from total RNA extracted from the lung tissue of infected hamsters by qPCR using a primer-probe set specific for the viral ORF1A/B region. Open circles represent female animals and closed circles males. (PDF) [file ppat.1010807.s003.pdf]

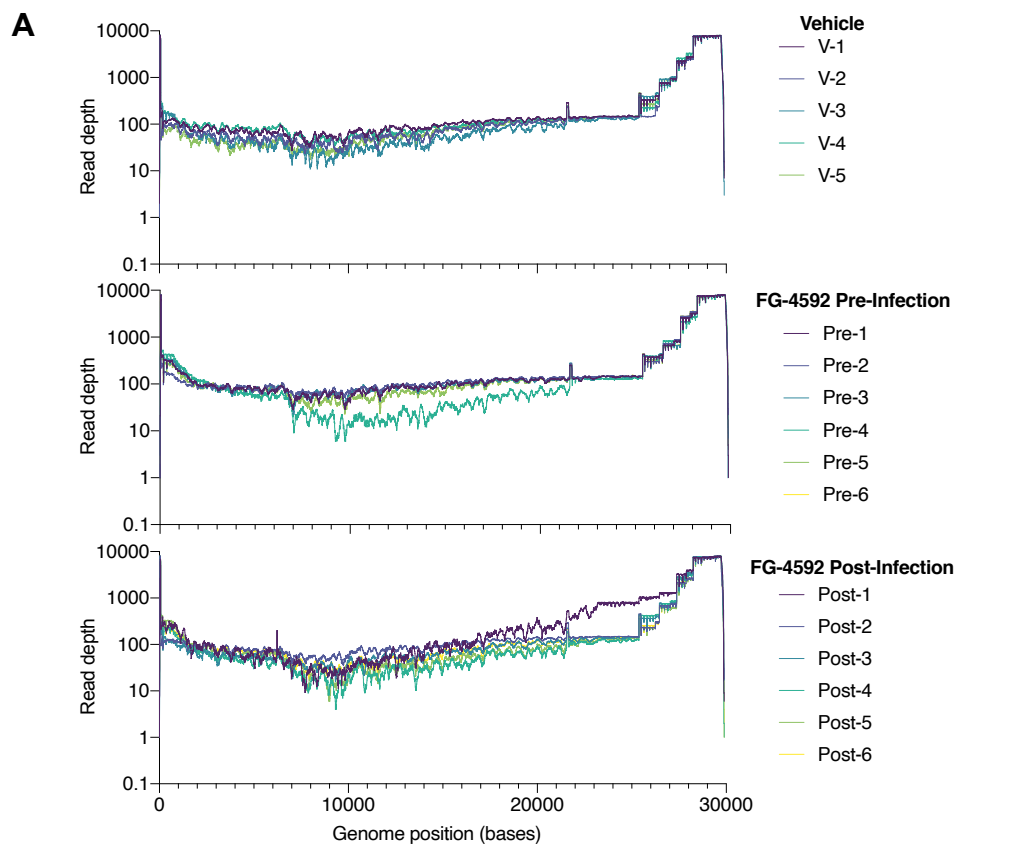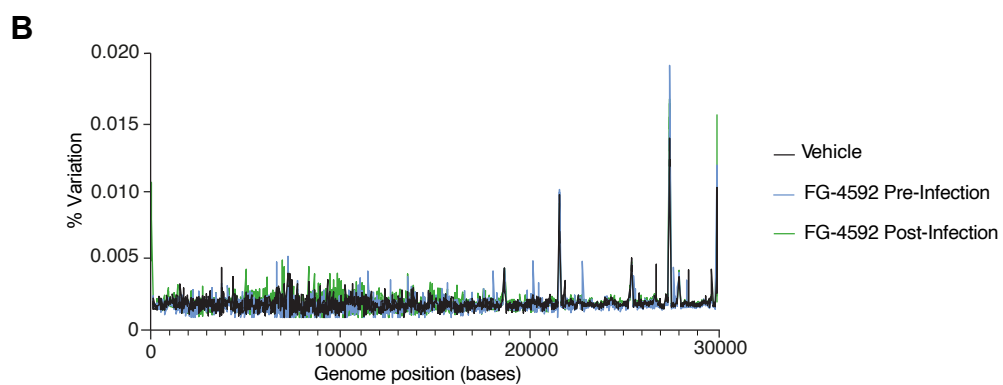

Supplement: S4 Fig — (A) SARS-CoV-2 viral reads were mapped to the genome using the SALMON alignment package and read depth at each position in the genome quantified. Samples from individual animals are grouped by the treatment they received. (B) Variability of the viral sequence across the treatment groups. At each nucleotide position the percentage of reads for each nucleotide (A, C, T, G) was divided by the read depth so that a value of 0 would represent 100% conservation across all reads. (PDF) [file ppat.1010807.s004.pdf]

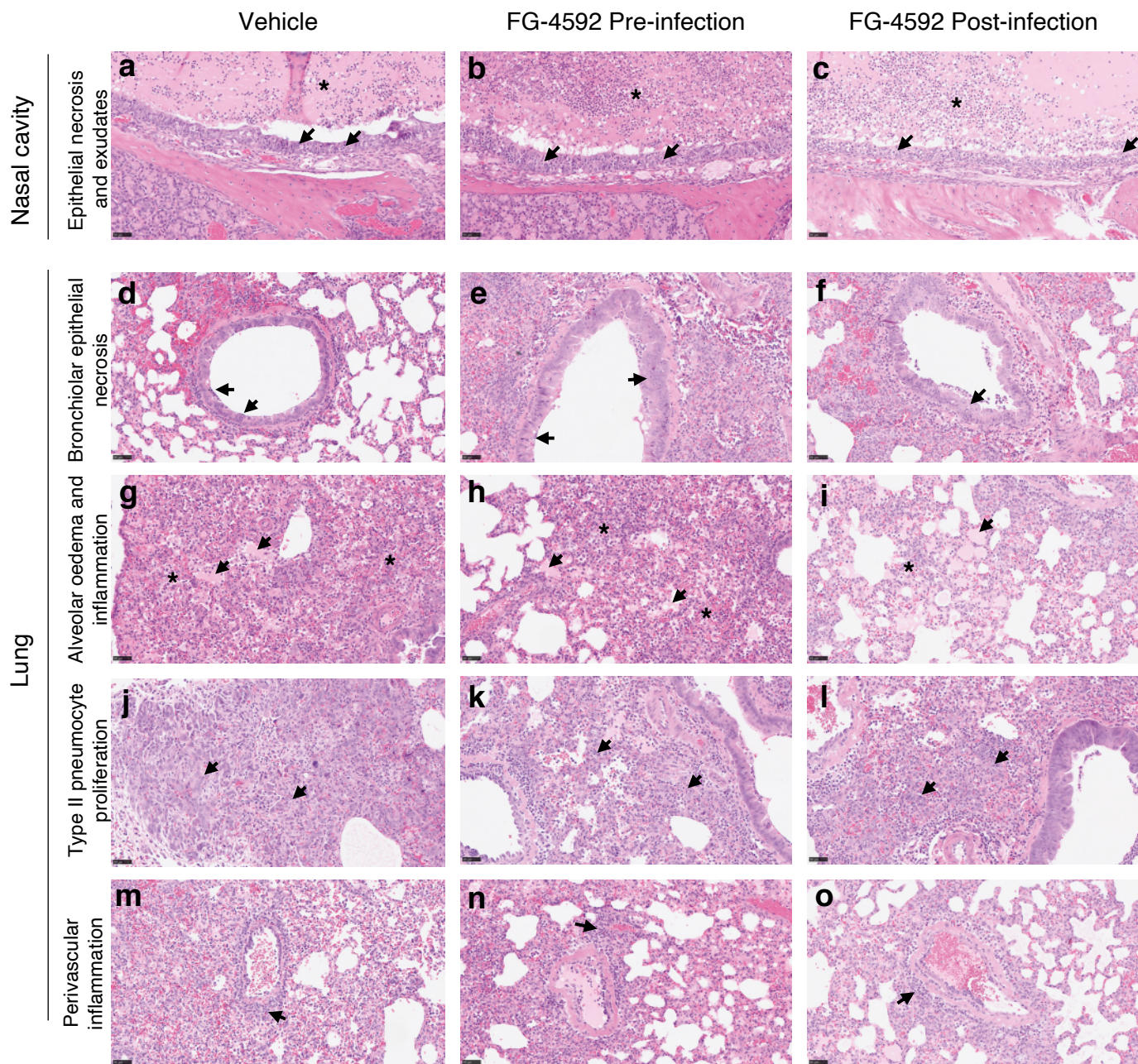

Supplement: S5 Fig — Histopathological changes observed in the nasal cavity (A-C) and lung (D-O). The main changes observed in the nasal cavity are presence of abundant mixed inflammatory exudates in the lumen (*) and mild to moderate epithelial cell necrosis (arrows) (A-C). Epithelial cell necrosis is observed in the bronchiolar epithelium (arrows) (D-F). Alveolar changes include presence of inflammatory cells within the alveolar spaces (*) and oedema (arrows) (G-I), together with type II pneumocyte proliferation (J-L; arrows). Perivascular cuffing with presence of inflammatory cells surrounding blood vessels (arrows) is observed (M-O). Scale bar = 50 μm. (PDF) [file ppat.1010807.s005.pdf]

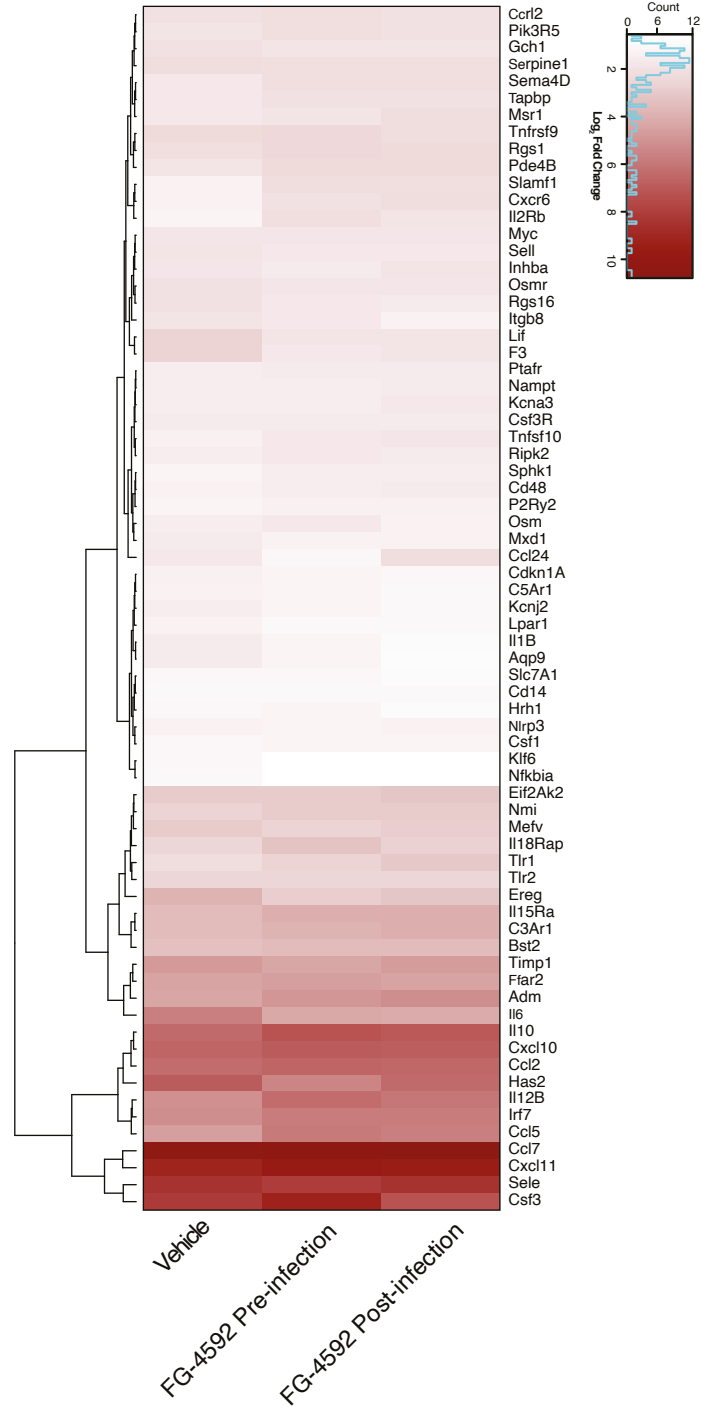

Supplement: S6 Fig — Expression of the inflammatory hallmark genes derived from the MSig database in vehicle, pre- and post-infection treatment with FG-4592 compared to uninfected samples. Hierarchical clustering was used to group genes according to expression. (PDF) [file ppat.1010807.s006.pdf]

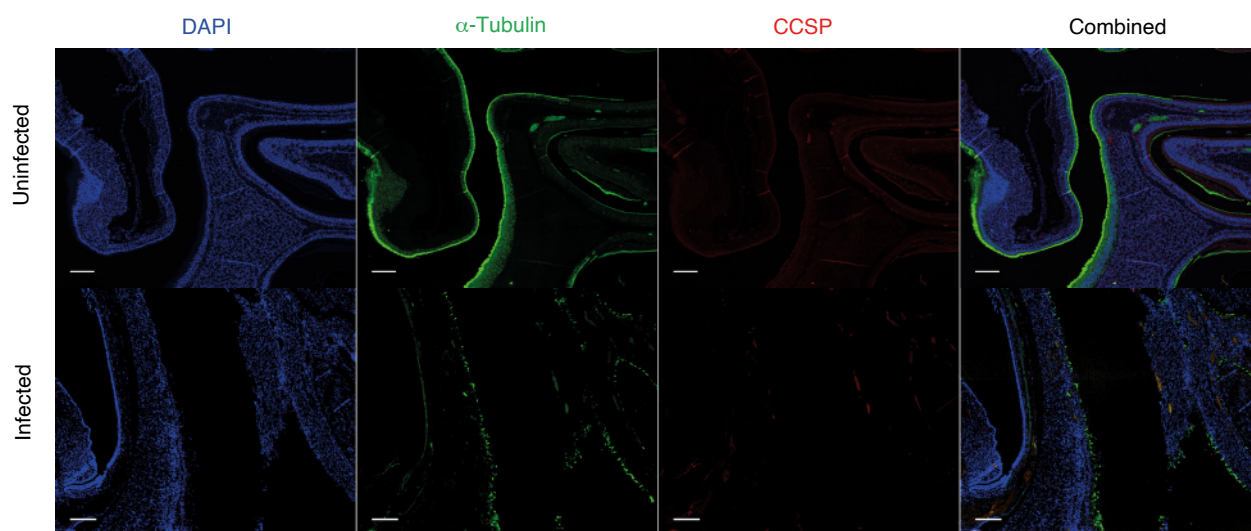

Supplement: S7 Fig — Representative nasal cavity sections from uninfected, vehicle, pre-infection, or post infection FG-4592 treated animals were stained by immunohistochemistry for a-tubulin and CCSP with nuclei visualised by DAPI. Individual stains are shown along with an overlayed image. Scale bars represent 100μm. (PDF) [file ppat.1010807.s007.pdf]

# Lung

Vehicle

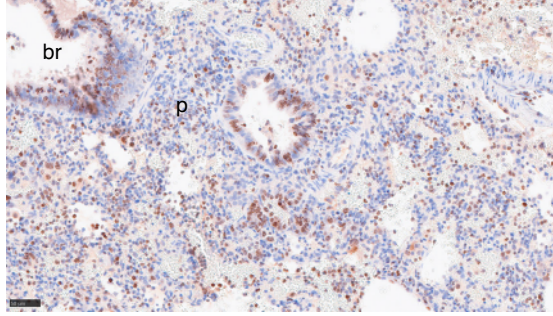

FG-4592 Pre-infection

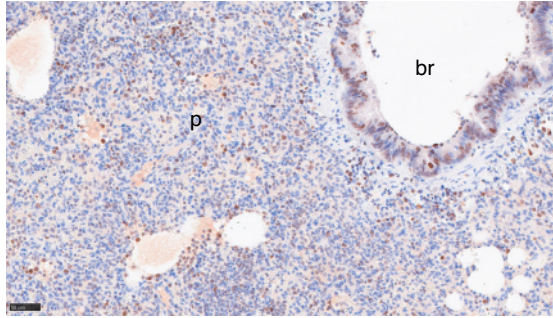

FG-4592 Post-infection

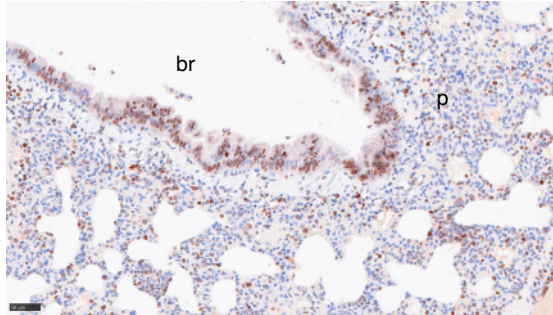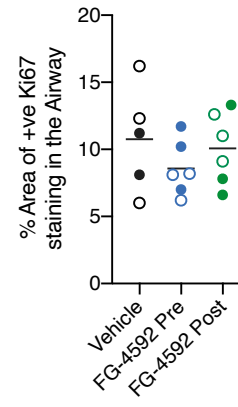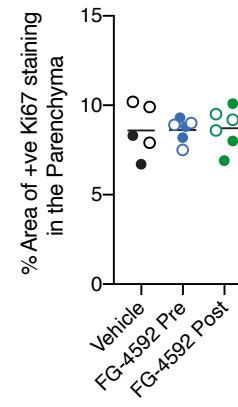

Supplement: S8 Fig — Lung sections from vehicle, pre- and post-infection FG-4592 treated animals were stained for Ki67 by immunohistochemistry. Br = bronchiole, p = parenchyma. Images were subjected to digital image analysis to quantify the amount of Ki67 staining as a percentage of the total area of either airway or parenchyma. Scale bar = 50 μm. Open circles represent female animals and closed circles males. (PDF) [file ppat.1010807.s008.pdf]
